# BRD4 facilitates DNA damage response and represses CBX5/Heterochromatin protein 1 (HP1)

**DOI:** 10.18632/oncotarget.17572

**Published:** 2017-05-03

**Authors:** Georgios Pongas, Marianne K. Kim, Dong J. Min, Carrie D. House, Elizabeth Jordan, Natasha Caplen, Sirisha Chakka, Joyce Ohiri, Michael J. Kruhlak, Christina M. Annunziata

**Affiliations:** ^1^ Women's Malignancies Branch, Center for Cancer Research, National Cancer Institute, Bethesda, MD 20892, USA; ^2^ Laboratory of Cancer Biology and Genetics, Center for Cancer Research, National Cancer Institute, Bethesda, MD 20892, USA; ^3^ Genetics Branch, Center for Cancer Research, National Cancer Institute, Bethesda, MD 20892, USA; ^4^ Experimental Immunology Branch, Center for Cancer Research, National Cancer Institute, Bethesda, MD 20892, USA

**Keywords:** heterochromatin, BRD4, CBX5, DNA repair, CHK1

## Abstract

Ovarian cancer (OC) is a heterogeneous disease characterized by defective DNA repair. Very few targets are universally expressed in the high grade serous (HGS) subtype. We previously identified that CHK1 was overexpressed in most of HGSOC. Here, we sought to understand the DNA damage response (DDR) to CHK1 inhibition and increase the anti-tumor activity of this pathway. We found BRD4 suppression either by siRNA or BRD4 inhibitor JQ1 enhanced the cytotoxicity of CHK1 inhibition. Interestingly, BRD4 was amplified and/or upregulated in a subset of HGSOC with statistical correlation to overall survival. BRD4 inhibition increased CBX5 (HP1α) level. CHK1 inhibitor induced DDR marker, γ-H2AX, but BRD4 suppression did not. Furthermore, nuclear localization of CBX5 and γ-H2AX was mutually exclusive in BRD4-and CHK1-inhibited cells, suggesting BRD4 facilitates DDR by repressing CBX5. Our results provide a strong rationale for clinical investigation of CHK1 and BRD4 co-inhibition, especially for HGSOC patients with BRD4 overexpression.

## INTRODUCTION

Ovarian cancer (OC) is the most aggressive gyneco-logical malignancy, with nearly 15,000 deaths in USA annually. Comprehensive genomic approaches identified molecular alterations in cancer, intended to enable precision medicine in which patients are selected for specific treatments based on molecular parameters. This strategy is expected to improve outcome for patients over empiric chemotherapy. The Cancer Genome Atlas (TCGA) analyzed samples from over 500 women with primary high-grade serous (HGS) OC, and found copy-number alteration as a major unifying characteristic [1]. Due to high genome instability and defective DNA damage repair (DDR), initial platinum chemotherapy is effective in most cases, but relapse within 18 months is common. Early relapse suggests that OC cells adapt to overcome DNA damage. Supporting this notion, we previously identified that checkpoint kinase 1 (CHEK1 or CHK1) was overexpressed in nearly all cases of HGSOC at the time of initial diagnosis, as compared to normal ovarian surface epithelium [2]. In HSGOC, p53 is either null or mutated, possibly increasing the cellular dependency on CHK1 for DDR and survival. LY2606368 is a potent CHK1 inhibitor (CHK1i) with anti-tumor activity [3, 4]. This agent is currently in clinical trials for many cancers, including our phase 2 study for women with HGSOC (NCT02203513).

Over-expressed CHK1 may be further activated in cells’ attempt to repair DNA upon exposure to standard chemotherapy. For example, we showed that topotecan (TPT), a salvage treatment for OC, activated CHK1, but that CHK1i reduced the concentration of TPT required to induce maximal cytotoxicity [5]. This suggested that CHK1 activation is a mechanism of resistance to TPT monotherapy, especially in CHK1-overexpressing HGSOC patients. We recently defined 55 candidate genes essential for OC survival from independent shRNA screens in 4 OC cell lines and further identified the potent combination of PLK1 and WEE1 inhibitors, providing a strategy to systematically refine therapeutic strategies in OC [6].

As a next step, we sought targets that sensitized OC cells to CHK1i and identified BRD4. Here, we describe a novel mechanism for BRD4 in the DDR process. We show that BRD4 represses expression of heterochromatin protein CBX5/HP1α. This may allow access for assembly of gamma-H2AX and DNA repair proteins. Pathologic BRD4 amplification, therefore, may foster unstable chromatin by enhancing DDR in the presence of overactive CHK1. BRD4 loss consequently restricts DDR and sensitizes to CHK1 inhibition. This provides rationale for a clinical strategy to treat patients whose tumors show BRD4 amplification and/or up-regulation.

## RESULTS

### BRD4 suppression increases death of OC cells with CHK1 inhibitor

**Figure 1:**
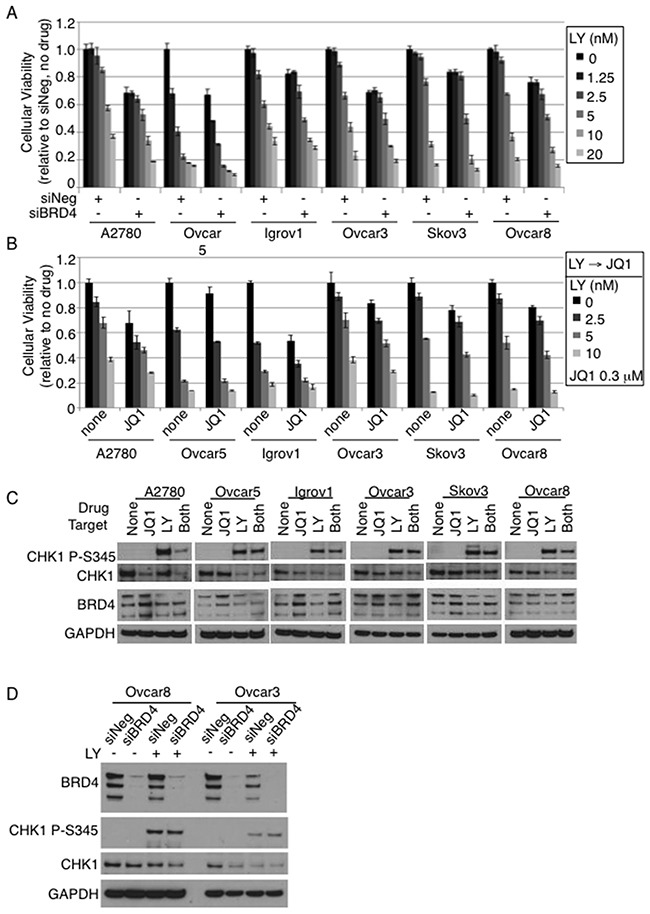
BRD4 suppression sensitizes OC cells to CHK1i **(A)** Cells transfected with either siNeg or siBRD4 were seeded at 2000 cells/well at 24hr post-transfection; drug was added after 24hr. Cell viability was measured after 48hr of drug treatment. Viability was calculated relative to no drug treatment; error bars represent standard deviation of 3 replicates. **(B)** Cells were seeded 24hr prior to adding LY2606368 and then JQ1 was added next day. Viability was measured 48hr after JQ1 treatment. **(C)** Cells were treated with JQ1 (2.5μM) and/or LY2606368 (2.5nM) for 24hr; total protein lysates were analyzed by Western blot. GAPDH was loading control. **(D)** siRNA transfected cells were treated with LY2606368 (10nM) for 16hr at 48hr post-transfection and harvested for total protein.

We first determined the viability of OC cells in the presence of CHK1i LY2606368 under conditions that we previously used to identify essential targets for OC survival (Supplementary Figure 1A) [6]. We finely tuned the concentration for LY2606368 to reach IC25 and IC50 in each cell line in combination with control siRNAs (Supplementary Figure 1B). LY2606368 IC25 and IC50 were determined to be 1.5–10 nM and 3-40 nM in the OC cell lines (Supplementary Figure 1C). Cellular viability with the combination of siRNA and CHK1i was measured in 5 OC cell lines; siRNA targeting I-kappaB kinase epsilon (IKBKE) was used as a positive control, based on our prior study that showed knockdown of IKBKE decreases viability in these cell lines (Supplementary Figure 1D) [2]. In our previous study, we found that siRNAs targeting BRD4, MAP3K7, and NLK increased the cytotoxicity of CHK1i in all 5 cell lines (Supplementary Table 1) [6]. Consistent with our previous screen, PLK1 loss was lethal in all cell lines tested regardless of CHK1i (Supplementary Figure 1D, Supplementary Table 1) [6]. We further focused on Bromodomain containing 4 (BRD4) due to its translational potential and confirmed this screening result by an independent siBRD4. In all 6 OC cell lines representing different subtypes of OC, cell viability was further decreased in siBRD4-transfected cells compared to siNeg in a LY2606368 dosage-dependent manner (Figure 1A). Effects were additive, and not synergistic. Of note, 3 isoforms of BRD4 were detected in all 6 cell lines with no correlation to CHK1 level (Supplementary Figure 2A). To further investigate the importance of BRD4 in the context of CHK1, we tested BRD inhibitor, JQ1 [7]. The IC50 ranged from approximately 0.1 – 2.5 μM with 3 days exposure (Supplementary Figure 2B). Consistent with the siBRD4, a sub-lethal concentration of JQ1 decreased the number of viable cells exposed to CHK1i regardless of the order of drug addition (Figure 1B, Supplementary Figure 2E-2F). Viability effects with chemical inhibition were similar to that of BRD4 knock down in that there was additive killing that was generally not synergistic. BRD4 protein level increased slightly upon JQ1 treatment (Figure 1C). Interestingly, BRD4 suppression either by siBRD4 or JQ1 did not trigger DNA damage response as measured by phosphorylation of CHK1 on Serine345, while either LY2606368 or the co-inhibition markedly increased CHK1 P-S345 and decreased total CHK1 (Figure 1C–1D). The subtle differences between cell lines may be due to molecular differences between the cell lines that could cause slight differences in patterns of protein expression. For example, A2780 has wild type p53 whereas Ovcar3, Ovcar8 and Igrov1 have mutant p53, and Ovcar5 and Skov3 are p53 null [2]. In addition, Igrov1 is a “hypermutated” cell line, and A2780 is not serous histology [8]. All of these molecular differences can influence expression of the proteins in response to CHK and BRD inhibition. While not identical, however, the results followed similar trends, suggesting a mechanistic relationship in ovarian cancer. Taken together, BRD4 suppression, either by siRNA or JQ1, adds to CHK1 inhibitor lethality in ovarian cancer cells.

### BRD4 is amplified in a subset of OC

**Figure 2:**
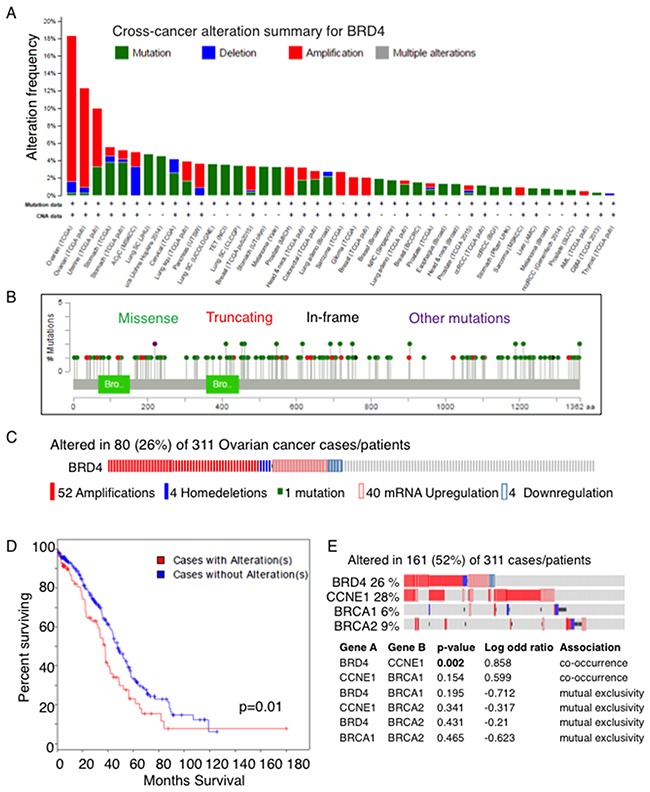
BRD4 is amplified and/or upregulated in OC **(A)** BRD4 alteration was examined in tumor samples using cBioPortal (www.cbioportal.org). BRD4 alterations occurred most commonly in ovarian cancer, shown by the alteration frequency in TCGA. Amplifications (red) were the most common alteration. **(B)** BRD4 mutations detected in TCGA tumors are shown. **(C)** BRD4 alterations including mutations, copy number, mRNA expression in TCGA OC dataset are shown. **(D)** Overall survival based on BRD4 alterations in TCGA OC is shown. **(E)** Mutual exclusivity of BRD4 alteration from TCGA data analysis among genes involved in OC.

In order to examine the clinical relevance of BRD4, we searched TCGA datasets. Across all cancer types, OC had the highest frequency of BRD4 amplification (Figure 2A). Other cancers including uterine, lung, pancreas, breast, prostate, sarcoma, and glioma showed lower frequencies of BRD4 amplification. Most mutations were missense throughout the entire protein without any specific hot spots (Figure 2B). With mRNA expression data included, the alteration frequency was 26% in OC: 52 cases with amplification, 40 with mRNA upregulation, 4 with mRNA downregulation, 1 with mutation, and 4 with deletion. (Figure 2C). These alterations were significantly associated with shorter overall survival compared to cases without alterations (p=0.01, Figure 2D). Interestingly, BRD4 alterations tended to co-occur with CCNE1 alterations (p=0.002), while there was a trend toward mutual exclusivity with BRCA1 or BRCA2 alterations (Figure 2E). The high frequency of BRD4 amplification and/or upregulation in OC and the correlation with poor survival suggest a pathologic role of BRD4 in OC tumorigenesis. Of note, none of the cell lines used in this study have BRD4 amplification (http://cancer.sanger.ac.uk/cell_lines).

### CBX5 is a repression target of BRD4

**Figure 3:**
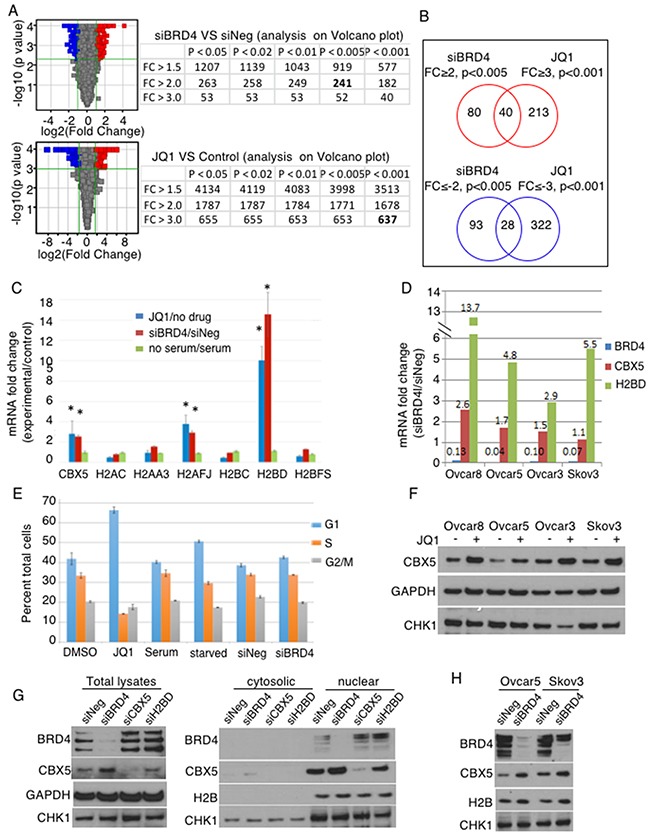
CBX5 is identified as a repression target of BRD4 **(A)** Differentially regulated genes in Ovcar8 cells were identified upon BRD4 knockdown and chemical inhibition by JQ1. The number of entities are shown with different cut-offs; numbers in bold are the entities used in B. **(B)** Up- and down-regulated genes are compared separately. **(C)** Candidate genes were validated by qPCR in Ovcar8 cells. Blue bar represents the average of 3 independent biological replicates of JQ1 treatment and red bar shows results from the siRNA transfection, and green bars represent serum starvation, as an experimental condition for non-specific cell cycle arrest. Each gene was normalized by GAPDH and compared to the negative control for each condition (starved/unstarved, siBRD4/siNEG, JQ1/DMSO). *, p<0.05 in ANOVA with Dunnet post-hoc correction for comparison of each to GAPDH. **(D)** Up-regulation of *CBX5* and *HIST1H2BD* genes was validated in 3 more OC cell lines upon BRD4 knockdown. Error bars represent standard error of the mean. ANOVA with Dunnett post-hoc showed all p-values <0.05. **(E)** Relative proportion of cells in each phase of the cell cycle was measured with propidium iodide under conditions of JQ1 treatment, serum starvation, or BRD4 knock down. Error bars represent standard error of the mean. **(F)** Ovcar8 cells were transfected with indicated siRNAs. Total protein lysates (30μg) or nuclear extracts (20μg) were analyzed by Western blot. GAPDH and H2B were loading controls. **(G)** Nuclear extracts were probed for changes after BRD4 knockdown, using H2B as a loading control. **(H)** Cells were treated with JQ1 (2.5 μM) for 24hr; total protein was analyzed by Western blot with GAPDH as loading control.

To further investigate molecular mechanisms of BRD4, we profiled gene expression upon BRD4 suppression (Figure 3A). Many more genes were differentially regulated with JQ1 compared to siBRD4, suggesting either more robust suppression or broader targeting to include BRD2 and BRD3. To prioritize candidate genes, we applied different stringency cut-off values to each set: absolute fold change (│fc│) ≥ 2 with p<0.005 for siBRD4 set and │fc│ ≥ 3 with p<0.001 for JQ1 set, and then compared the up- and down-regulated genes separately to identify common genes (Figure 3B, Supplementary Table 2). Curiously, many genes regulating chromatin structure, including histone genes and *CBX5 (HP1α)*, were commonly up-regulated upon BRD4 suppression. By qPCR, *HIST1H2BD* and *CBX5* were consistently validated with both JQ1 and siBRD4 (Figure 3C). We confirmed this in additional OC cell lines by siBRD4 knockdown (Figure 3D, Supplementary Figure 3). The data do not distinguish between mRNA expression and 3’ end processing because the method used to isolate RNA could have lost transcripts with altered polyA tails. In order to determine whether this was a general effect due to cell cycle arrest, we induced G1 arrest by serum starvation. Cell cycle arrest by serum starvation did not induce the histone genes, whereas the knockdown of BRD4 by siRNA or inhibition by JQ1 induces expression of *CBX5*, *H2AFJ*, and *HIST1H2B* (Figure 3C). Cell cycle arrest was measured by propidium iodide staining under conditions of JQ1 exposure, serum starvation, and BRD4 knockdown. The most dramatic G1 arrest was achieved by JQ1 exposure (Figure 3E). Serum starvation also induced cell cycle arrest, as did BRD4 knockdown to a lesser extent. These results suggest that the induction of *CBX5* and histone mRNA was not solely due to arrest of the cell cycle. This was further examined at the protein level (Figure 3F–3H). We were unable to detect changes in HIST1H2BD protein despite prominent changes at the mRNA level (Supplementary Figure 3, and data not shown). Because of the particular susceptibility of histone gene transcription to mRNA 3’end processing, and our inability to detect changes at the protein level, we did not further pursue alteration in histone genes, but instead focused on CBX5. The increase in CBX5 was detected upon JQ1 treatment in all 4 cell lines tested (Figure 3F). The increase in CBX5 protein level upon BRD4 knockdown was consistently observed in either total protein or nuclear lysate fractions. BRD4 knockdown did not affect CHK1 level, while JQ1 decreased in CHK1 especially in Ovcar3. This difference between RNAi knockdown and chemical inhibition of BRD proteins may be due to the effect of JQ1 on BRD proteins other than BRD4. In addition, the complete loss of BRD4 protein may affect other non-enzymatic functions of BRD4 (i.e., bromodomain-acetyllysine-independent functions) that are not inhibited by JQ1. Overall, however, the effect of BRD4 inhibition by either RNAi or JQ1 was consistent in inducing CBX5 expression at both mRNA and protein levels.

**Figure 4:**
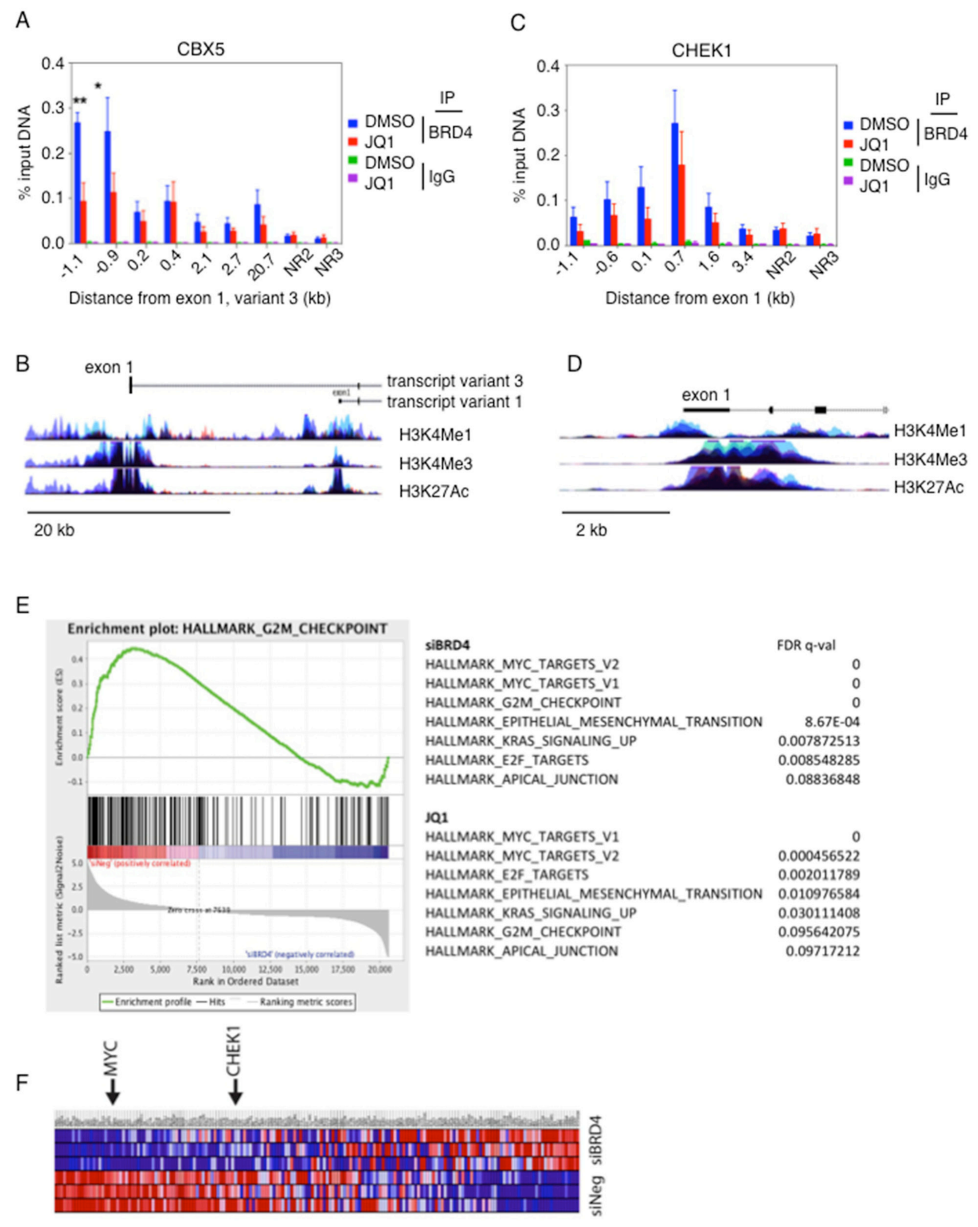
BRD4 directly interacts with the promoter region of CBX5 **(A)** OC cell line Ovcar8 was treated with JQ1 and chromatin was immunoprecipitated with anti-BRD4 antibody. Quantitative PCR detected regions of chromatin that were precipitated with the BRD4 antibody. **(B)** The genomic region of BRD4 is shown, marking the region surrounding exon 1 start site, NR2 and NR3 are negative control regions that are found 1Mb distant from the MYC promoter. **(C)** Chromatin from Ovcar8 cells was precipitated under the same conditions and quantitative PCR for genomic loci in the region of CHK1 was performed. For A and C, error bars represent standard error of the mean. **(D)** Schematic representation indicates the location of amplified regions, relative to the start of exon 1. **(E)** Gene set enrichment analysis identified G2M checkpoint as a top signature regulated by BRD4 in Ovcar8 cells. **(F)** Genes in the G2M checkpoint signature showed altered expression by BRD4 knockdown. MYC and CHK1 are included in this signature. Red indicates upregulation and blue indicates down regulated genes.

We proceeded to perform chromatin immuno-precipitation of BRD4 under conditions of JQ1 exposure in order to determine whether BRD4 directly interacted with the DNA immediately upstream of *CBX5* (Figure 4A). The enrichment of precipitated DNA at approximately 1000b upstream of exon 1 of the *CBX5* gene indicates that BRD4 directly binds close to the CBX5 gene (Figure 4B). This binding decreased upon exposure to JQ1. The interaction was specific to the CBX5 gene, in that there was no binding to negative control regions (NR2 and NR3, 1 Mb upstream of MYC promoter) and there was no non-specific enrichment of the IgG control antibody in any of the chromatin tested. Interestingly, BRD4 also appeared to interact with the first exon of *CHK1* gene (Figure 4C–4D). This binding was slightly decreased with JQ1 treatment, consistent with the slight changes observed in protein expression in some cell lines (Figure 1C).

Using gene set enrichment analysis of expression profiles following BRD4 knock down, we identified the MYC signature as one of the top networks regulated by knockdown of BRD4, consistent with previously published data [9]. Interestingly, another top network was the hallmark G2M checkpoint signature (Figure 4E). This is consistent with our findings of G2M cell cycle decrease with BRD4 knockdown (Figure 3E). Both *MYC* and CHK1 were within the genes identified as down regulated in this signature (Figure 4F). These results corroborate effects of cell cycle arrest rather than apoptosis caused by attenuation of BRD4.

### BRD4 prevents heterochromatin and allows DNA damage response

**Figure 5:**
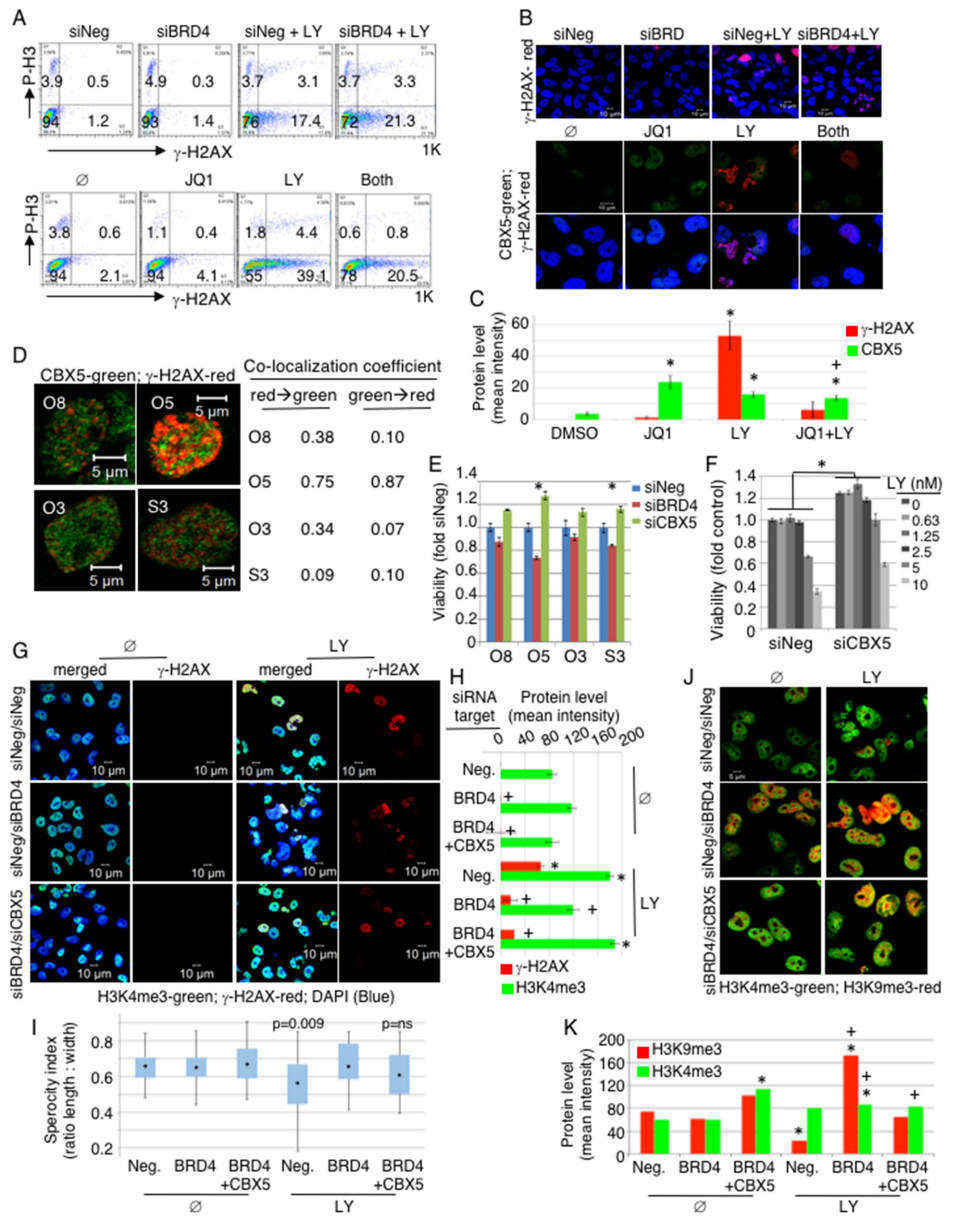
BRD4 suppression induces heterochromatin, inhibiting DNA damage response **(A-D)** Ovcar8 cells were transfected with either siNeg or siBRD4; 24hr later the cells were treated with 5nM LY for 20hr. For chemical inhibition, cells were treated with JQ1 (2.5μM) and/or LY (10 nM) for 20hr and analyzed by flow cytometry (A), or for 30hr in immunofluorescent staining (B-D). Resulting images from (B) were quantified in (C). By ANOVA with Tukey post-hoc: *, p<0.05 comparing to DMSO control; +, p<0.05 comparing to JQ1 alone. Samples in (D) received both JQ1 (2.5μM) and LY (10nM). **(E)** Cells were transfected with indicated siRNA at a final concentration of 20nM and 24hr later seeded in 3 replicates. Viability was measured by XTT and normalized to siNeg transfected cells. Results were statistically significant in Ovcar5 and Skov3 (ANOVA with Dunnett post-hoc, p<0.05, indicated by asterisk). **(F)** Ovcar8 cells were transfected with either siNeg or siCBX5 and 24hr later seeded in 3 replicates. Drug was added 24hr after plating. Viability was measured by CellTiter Glo and normalized to untreated siNeg cells. Differences with CBX5 knock down were significantly different at all concentrations of LY (ANOVA with Tukey post-hoc, p<0.001). **(G-K)** Ovcar8 cells were transfected with 20nM indicated siRNAs. For co-transfection, 10nM of each siRNA was added for the same total siRNA concentration in each sample. At 24hr post-transfection, cells were plated on cover slips and 8hr later LY (10nM) was added and further incubated for 24hr before immunostaining. Multiple fields were analyzed, containing 10-40 cells each. (G) Merged images of H3K4me3, γ-H2AX and DAPI are shown in the left column, and γ-H2AX alone is shown in the right column of untreated (Ø) and LY treated panel. (H) Staining intensity for γ-H2AX and H2K4me3 was measured. Error bars represent standard error of the mean. By ANOVA with Tukey post-hoc: *, p<0.05 comparing to Neg/Ø control; +, p<0.05 comparing to Neg/LY control. (I) Sphericity was estimated for cells in (G) by calculating the ratio of two perpendicular diameters of individual cells. ANOVA with Dunnett adjustment indicated loss of nuclear roundness (p=0.09). (J) Cells were co-stained with H3K4me3 and H3K9me3 antibodies. (K) Immunofluoresecence for H2K9me3 and H2K4me3 was quantified for each experimental condition. By ANOVA with Tukey post-hoc: *, p<0.05 comparing to Neg/Ø control; +, p<0.05 between conditions with or without LY.

We next investigated potential mechanisms underlying the functional relationship between BRD4 and CHK1. Upon DNA damage, CHK1 is activated to repair DNA, and its inhibition allows premature entry into mitosis with un-repaired DNA, resulting in mitotic catastrophe and subsequent cancer cell death. We tested whether BRD4 inhibition would accelerate mitotic catastrophe by enhancing DNA damage. Phosphorylation of histone H3 protein occurs upon entry into mitosis, and phosphorylation of histone H2 occurs with DNA damage. We examined changes in the mitotic marker phospho-histone H3 (P-H3) and DNA damage marker phospho-H2AX (γ-H2AX) upon BRD4 suppression in the absence and presence of LY2606368. Flow cytometry showed no significant changes in P-H3 upon either BRD4 knockdown or LY2606368 treatment, suggesting that BRD4 does not regulate mitotic entry (Figure 5A, Supplementary Figure 4A-4B). Prominent increases in γ-H2AX upon LY2606368 treatment were observed in Ovcar8, Ovcar5, A2780, and Skov3 as expected. Interestingly, there were no changes in γ-H2AX upon BRD4 suppression either by siBRD4 or using JQ1. Therefore, it is unlikely that BRD4 inhibition directly promotes DNA damage, as also shown by lack of CHK1 phosphorylation at S345 (Figure 1C). Immunofluorescent (IF) staining also showed that BRD4 knockdown or JQ1 exposure did not increase the amount of phosphorylated γ-H2AX in the nucleus (Figure 5B, Supplementary Figure 4C). On the contrary, we observed decreased level of γ-H2AX upon co-inhibition of BRD4 and CHK1 compared to CHK1 inhibition alone.

By these same measures, CBX5 (HP1α) expression increased upon BRD4 knockdown (Supplementary Figure 4D). CBX5 is a protein known to mark condensed heterochromatin during interphase. Heterochromatin is not easily accessible to the DNA repair machinery [10]. CBX5 reproducibly increased in all 3 cell lines upon JQ1 treatment, and γ-H2AX increased with LY2606368 treatment alone as well as the co-treatment (Figure 5B, lower; Supplementary Figure 4E). CBX5 staining increased with JQ1 exposure, and γ-H2AX increased with LY treatment (Figure 5C). Notably, the rise in γ-H2AX was attenuated by combined BRD4 and CHEK1 blockade, but there was a discrepancy between gene knock down of BRD4 versus chemical inhibition of BRD4 with JQ1, especially in reference to γ-H2AX. In the setting of gene knock down, the combined effect of siBRD4 and LY treatment increased γ-H2AX. In the setting of chemical inhibition, the combined JQ1 and LY decreased γ-H2AX compared to LY alone, on both flow cytometry and immunofluorescence. This may be due to the biological difference between decreasing the entire protein versus inhibiting its chemical function, while leaving possibly non-enzymatic scaffolding functions intact. In addition, the chemical JQ1 is known to inhibit BRD2 and BRD3 in addition to BRD4, and these other proteins may provide additional influence on γ-H2AX phosphorylation.

Importantly, localization of CBX5 and γ-H2AX was mutually exclusive in the nucleus, indicating that DDR did not occur in regions of condensed heterochromatin (Figure 5D). We quantified the co-localization of CBX5 and γ-H2AX, and found a low co-localization coefficient, indicating that these two proteins predominantly bind distinct DNA loci (Figure 5D). More interestingly, knockdown of CBX5 enhanced cellular viability and rendered cells more resistant to CHK1i (Figure 5E–5F). This suggests that BRD4 supports OC survival by suppressing CBX5 expression and allowing DNA repair machinery access to damaged DNA. If this holds true, then knockdown of CBX5 should rescue the phenotype of BRD4 loss. We tested this hypothesis by transfecting cells with siNeg, siNeg+siBRD4, or siBRD4+siCBX5. In the absence of CHK1 inhibition, there were no significant changes in nuclear morphology or changes in γ-H2AX under these conditions (Figure 5G, Supplementary Figure 4F). CHK1 inhibition caused abnormal nuclear morphology with increased γ-H2AX staining. Quantification of trimethylated histone H3 at the K4 locus, a marker of euchromatin, showed a decrease in euchromatin with BRD4 knock down. Consistent with our hypothesis, this was rescued when CBX5 was co-depleted with BRD4 in cells treated with LY (Figure 5H). We estimated “sphericity” as a description of nuclear morphology, by measuring two perpendicular dimensions of each cell. These data show a strong loss of nuclear roundness with LY exposure (p=0.009) that was completely restored by knock down of CBX5 (Figure 5I). These results are consistent with the hypothesis that chromatin dysregulation after CHEK1 and BRD4 inhibition is mediated by CBX5. We also investigated expression of euchromatin (H3K4me3) and heterochromatin (H3K9me3) markers (Figure 5J, Supplementary Figure 4G). BRD4-depleted cells showed increased H3K9me3 that was reversed by knocking down CBX5, especially in the presence of CHK1 inhibition. We further quantified the immunofluorescence of euchromatin marker H3K4me3 and heterchromatin marker H3K9me3. Notably, there was an increase of H3K9 methlylation with BRD4 knock down in cells exposed to LY. This again was largely abrogated by knock down of CBX5 (Figure 5K). In independent experiments, we reproducibly found a significant increase in H3K9me3 only in the siBRD4 transfected sample. These data suggest that BRD4 loss induced heterochromatin structure, and the concurrent loss of CBX5 reversed the consequence of BRD4 loss (Supplementary Figure 4G). Similar trends were seen by Western blot of nuclear protein (Supplementary Figure 4H). Taken together, these results indicate that BRD4 supports DNA repair by preventing H3K9 methylation.

## DISCUSSION

Functional genomics and TCGA data uncovered a novel pro-survival mechanism orchestrated by BRD4 and CHK1 in OC. Like other clinically relevant CHK1 inhibitors, LY2606368 causes impaired DNA synthesis and premature entry into mitosis [3, 11]. Here we report a novel mechanism by which BRD4 promotes DNA repair: it represses the expression of heterochromatin protein CBX5/HP1α, implying an active role of BRD4 to maintain open chromatin structure to allow repair of damaged DNA. BRD4 is amplified and/or upregulated in a subset of HGSOC with significant correlation to poor survival. BRD4 loss sensitized OC cells to CHK1i, suggesting a biomarker-driven targeted approach to treatment of this deadly disease.

Overexpression of CHK1 may allow cells to tolerate the stress of an unstable genome. The dependency on CHK1 for recognition and repair of DNA damage may be critical in HGSOC cells containing dysfunctional p53 and high genomic instability. Hence, it is plausible that amplification or upregulation of BRD4 is an additional oncogenic mechanism to coordinate the pathological action of CHK1 by maintaining open chromatin structure through repression of CBX5. Upon BRD4 loss, increased CBX5 caused condensed chromatin structure, and this persistent condensation may inhibit downstream DNA damage recognition and repair leading to cell death.

BRD4 is of interest as a therapeutic target in hematological and solid tumors including ovarian cancer [12–19]. In OC, dependence on BRD4 was implied by sensitivity to inhibition by JQ1 based on MYCN expression [16]. The antitumor effect of JQ1 through MYC repression was also shown in neuroblastoma, medulloblastoma, hepatocellular carcinoma, and acute myeloid leukemia [9, 19–21]. Sustained BRD4 suppression may disrupt homeostasis of normal tissue, implying potential adverse outcome associated with BRD4 inhibition in patients [22]. Our data show that combined inhibition of BRD4 and CHK1 produces maximal cytotoxic effects at reduced dosages, potentially reducing side effects of either drug and increasing the therapeutic index for cancer treatment.

Chromatin condensation can influence sensitivity to DNA-damaging agents. Less compacted chromatin is vulnerable to DNA double stand breaks by gamma-radiation [23]. Furthermore, heterochromatin is generally resistant to γ-H2AX foci formation and DDR, and γ-H2AX modification occurs mostly in euchromatin [10, 24]. However, dynamic chromatin structure may be integral to DDR whereby transient chromatin compaction actually induces downstream DDR but persistent condensation inhibits DNA repair [25]. Indeed, an alternative role of BRD4 in the regulation of DDR has been proposed whereby BRD4 may suppress DDR independently at the transcriptional level by the direct recruitment of condensin II complex and subsequent condensation of chromatin [26].

Taken together, CHK1 facilitates DNA damage repair in open chromatin structure maintained by BRD4 allowing genomically unstable OC cells to repair intrinsic and extrinsic DNA damage. BRD4 suppression induced heterochromatin structure protein CBX5/HP1α, limiting DDR and thereby sensitizing OC cells to CHK1i leading to cellular death. Recurrent ovarian cancer is a uniformly fatal disease. Targeted therapies as single agents have had minimal effect on prolonging life in women with recurrent ovarian cancer, except in those with known susceptibility, such as BRCA mutation. Single agent treatments typically induce short remissions, if any, and relapse is unavoidable. Our ongoing experience with the CHK1 inhibitor, prexasertib shows promise in some but not all women with recurrent high grade serous ovarian cancer (NCT02203513). It is critical to identify targeted agents that combine to increase killing of ovarian cancer, and our previous screen identified this combination as particularly effective. Our study provides strong rationale for clinical investigation of this combination especially in women whose cancers overexpress both CHK1 and BRD4.

## MATERIALS AND METHODS

### Cell lines

All OC cell lines in this study were previously described, and maintained in RPMI supplemented with 10% heat-inactivated FBS [27].

### Chemical inhibitors

Stock solutions of JQ1 (Selleck, S7110) and LY2606368 (Lilly Oncology) were prepared in DMSO aliquots stored at -80°C. LY2606368 was provided under NCI MTA ref. no 37817.

### siRNA screen and transfection

siRNA conditions were described previously [6]. SMARTpool ON-TARGETplus siRNAs were purchased from Dharmacon (siNeg, D001810; siBRD4, L004937; siCBX5, L004296; siHIST1H2BD, L013137) for validation experiments. Transfection was performed at final concentration 20nM using DharmaFECT1 Transfection Reagent (GE Dharmacon, T-2001-01).

### Viability assay

Cells were seeded in 96-well plates at 2,000-4000 cells/50μl/well in triplicate. Drug in 50μl was added 24hr after seeding and either CellTiter Glo (Promega) or XTT (Sigma) assay performed 3 days after drug treatment unless indicated. Cell proliferation was calculated relative to negative control and standard deviation was calculated from triplicates. IC50 values were calculated from the individual data points obtained from XTT assay, using Compusyn software. The algorithm used in Compusyn is the method of Chou. Briefly, the median effect dose (Dm) is obtained from the anti-log of the x-intercept of the median effect plot: log(Fa/Fu) = m*log(D) - m*log(Dm) where Fa is Fraction affected, Fu is Fraction unaffected, m is slope [28].

### The Cancer Genome Atlas data

TCGA ovarian cancer dataset was analyzed using web-based tool: http://www.cbioportal.org [29, 30].

### Microarray analysis

Ovcar8 cells were transfected with either siNeg or siBRD4 in 3 independent plates and harvested 48hr post-transfection. For chemical inhibition, JQ1 was added for 24hr at final concentration 0.5μM in Ovcar8; DMSO was used for control in 3 independent plates. Total RNA was isolated using RNeasy with on-column DNase treatment (Qiagen). Microarray analysis was previously described [2, 6]. Briefly, samples were prepared, labeled and hybridized to Affymetrix H133Plus2.0 gene chips and scanned on Affymetrix GeneChip 3000 (Affymetrix, Santa Clara, CA). CEL files were imported into GeneSpring (Agilent); probe levels were normalized by GC-RMA algorithm, and entities with intensity values <100 were filtered out before performing significance analysis. Gene set enrichment analysis (GSEA) was performed in the drug treated group and the siRNA group. Hallmark signatures shared in common between these two groups with false discovery rate (FDR) <0.1 [31].

### Quantitative PCR

Ovcar8 cells were treated for 48h with 6 different conditions including 1. DMSO 2. JQ1 2.5uM 3. Regular medium (RPMI with 10% FBS) 4. Optimem medium 5. siRNA neg control 6. siRNA BRD4. Experiment was done three times. Total RNA (1μg) was converted to cDNA using iScript cDNA Synthesis Kit (Bio-Rad, # 170-8890) in 20μl reaction, which uses both oligo(dT) and random hexamers to capture RNA species. cDNA was diluted 1:5 in H_2_O and 2μl used in each 20μl real-time PCR reaction (QuantiTect SYBR Green PCR Kit, Qiagen, #204143). Quantification was performed in triplicate by 7900HT Fast Real-Time PCR System (Applied Biosystems). Each mRNA expression level was normalized to GAPDH. QuantiTect Primers were purchased from Qiagen: BRD4 (QT00044345), CBX5 (QT00045283), HIST1H2AC (QT00233590), HIST2H2AA3 (QT00235851), H2AFJ (QT01022798), HIST1H2BC (QT00243495), HIST1H2BD (QT00022813), H2BFS (QT00227199). Each experimental condition was normalized to the housekeeping gene (GAPDH) that was run independently in each sample, and then the result normalized to the untreated sample for each gene analyzed. The serum starved condition was compared to the un-starved. In the BRD4 set, the expression of each gene under the knockdown condition (siBRD4) was normalized to the expression of the gene when negative control siRNA (siNeg) was present. In the JQ1 set, the expression of the indicated gene was normalized between samples that were treated with JQ1 or with vehicle control (DMSO).

### Western blot analysis

Total protein was extracted from sub-confluent cells with 1% NP40 lysis buffer containing 150mM NaCl, 50mM TrisHCl, 10% glycerol, 1X Halt proteinase inhibitor cocktail, 5mM NaF, and 1mM NaOrthovanadate. For nuclear lysate preparation, nuclear complex Co-IP kit (Active Motif, #54001) was used. Protein concentrations were estimated using BCA Protein Assay Kit (Thermo Scientific, Rockford, IL). Antibodies cleaved-PARP (Cell Signaling, #9541), BRD4 (Cell Signaling, #13440), HP1α/CBX5 (Cell Signaling, #2616), H2B (Millipore, 07-371), H2BD (Thermo Scientific, PA5-30561), Phospho-CHK1 (Ser345) (Cell Signaling, #2348), CHK1 (Santa Cruz, sc-8408), and GAPDH (Millipore, MAB374), and secondary antibodies ECL anti-rabbit IgG HRP and ECL anti-mouse IgG HRP (GE Healthcare) were used at 1:5000 dilutions. The band was visualized using either Lumina Classico or Crescendo Western HRP substrate system (Millipore) depending on signal intensities. Experiments were repeated three times; shown are representative blots for each.

### Flow cytometry

siRNA transfected cells were treated with 5nM LY2606368 at 48hr post-transfection for 20hr. For JQ1 and LY2606368, cells were seeded and serum-starved for 16hr before adding 2.5μM JQ1 and/or 10nM LY for 20hr. Cells were fixed in 4% paraformaldehyde/PBS for 15min at room temperature (RT), washed twice with PBS, and then permeabilized with 0.25% TritonX100/PBS for 5min at RT. Cells were blocked in 10% goat serum/PBS for 30min at RT, incubated with primary antibodies for 2hr and then secondary antibodies for 1hr at 4°C. P-H3 (Ser10) (Cell Signaling, #9706) and γ-H2AX (Ser139/Tyr142) (Cell Signaling, #5438) were diluted in 10% goat serum/PBS and used at 1:50 and 1:200, respectively. AlexaFluor 488 conjugated-goat anti-rabbit (A11034) and AlexaFluor 647 conjugated-goat anti-mouse (A21236) antibodies (Molecular Probes) were used at 1:500. Cells were analyzed by FACS Calibur (Becton Dickinson) and quantified using FlowJo software.

### Cell cycle

For the single drug, serum starvation or siRNA experiments, Ovcar8 cells were seeded overnight at the 1×10^5^ cells/well of a 6 well plate. The next day, medium was changed and Ovcar8 growing cells were treated for 24h with 6 different conditions including 1. DMSO 2. JQ1 2.5uM 3. Regular medium (RPMI with 10% FBS) 4. Optimem medium (serum starvation) 5. siRNA neg control 6. siRNA BRD4. Cells were harvested with trypsin, washed one time with ice cold FACS buffer (PBS with 2%FBS) and resuspended in 1ml ice cold 70% ethanol and stored at 4°C overnight. Next day, the samples were transferred to -20°C and stored until the day of reading. The day of reading, cells were washed two times with 1ml FACS buffer and resuspended in 1ml of staining buffer [PBS, 1xPropidium Iodide and RNAse A (Invitrogen)] for 1h at 37°C. Cells were analyzed by FACS Calibur or FACS Canto II. Cell cycle was analyzed using FlowJo software. For the drug combination experiments Ovca8, Ovcar5, Skov3 and A2780 cells were seeded overnight at 0.5×10^5^ cells/ml. The next day, cells were treated for 24h with DMSO, JQ1 2.5uM, LY2606368 10nM or combination of JQ1 with LY2606378. Same methodology as prementioned was followed for the cell cycle analysis.

### Chromatin immunoprecipitation

Ovcar8 (~1×10^7^) growing cells were treated for 24h with DMSO or 500nM JQ1. Cells were cross-linked with 1% formaldehyde for 10 min at RT. Cross-linking was quenched with 125mM glycine for 10min at RT. Cells were rinsed twice with ice cold PBS, and scraped while on PBS with 1x Protease inhibitor (539134 Protease Inhibitor Cocktail Set III, EDTA-Free - Calbiochem) and 1mM PMSF. Cells were collected, centrifuged and resuspended in ice-cold RIPA (10 mM Tris HCl pH 8, 140 mM NaCl, 1mM EDTA pH8, 0.5mM EGTA, 1% Triton X-100, 0.1% sodium deoxycholate, 0.3% SDS, 1x Protease inhibitor, 1mM PMSF). DNA was sheared with Fisher Scientific FB-120 at an amplitude of 25%, performing 10×20s sonication cycles with 59s intervals between each cycle. Each immunoprecipitation sample, containing approximately 5 × 10^6^ cells was incubated overnight at 40C with 1ug of IgG control antibody (Santa Cruz; catalog no. 2027) or BRD4 antibody (Bethyl; catalog no. A301-985A). The next day, 25ul of protein G magnetic beads (Invitrogen) were incubated with each immunoprecipitation sample for 4h at 4oC. The samples were washed 3 times with ice cold RIPA (10 mM Tris HCl pH 8, 140 mM NaCl, 1mM EDTA pH8, 0.5mM EGTA, 1% Triton X-100, 0.1% sodium deoxycholate, 0.1% SDS, 1x Protease inhibitor, 1mM PMSF), one time with LiCl Buffer (10mM Tris HCl, pH8, 250mM LiCl, 0.5% NP40, 0.5% Sodium Deoxycholate, 1mM EDTA), one time with TE buffer (pH8) and resuspended in 100ul TE(pH8). Each chromatin precipitation sample and 50ul from the total chromatin samples were incubated with 20ug and 40ug of RNAse A (Invitrogen) respectively, overnight at 65°C. The next day the chromatin precipitation sample were resuspended at RIPA with 0.3% SDS. The chromatin precipitation sample and the total chromatin samples were treated with 20ug and 40ug of proteinase K (Invitrogen), respectively for 3h at 50°C. DNA was purified with QIAquick columns.

### Chip-Quantitative PCR

The sequences of the primers that were used for the chip-qPCR were the following:

Negative Controls (gene desert aproximately 1Mb upstream the myc)

**Table:** 

NR2	TCCTGGGTAGGAACCAGTTG	Forward
	ACTCACCAAGAGCTCCTCCA	Reverse
NR3	AAGCCAACCCATCACTGAAC	Forward
	TTCCCATGTTACCCACCACT	Reverse

CBX5 (kb relative to 1st exon of 3rd variant_NM_012117.2)

**Table:** 

-1.1	GTGCTAGGAAATCGCAAGCG	Forward
	CATTGTGGAGATGCACCCCT	Reverse
-0.9	AACTCATGGTTGGCGGAGAG	Forward
	TGCACCAGCCCATTCTACAG	Reverse
0.2	GACTCCATTTGGGCCCGTTA	Forward
	TTCTATTGGTTGGCCCGACC	Reverse
0.4	TTCTGAGCAGCGTCTCACTG	Forward
	TCCCCATTGGAGTCAAACCG	Reverse
2.1	GGAGTATGCAGGGCACAGTT	Forward
	TCTCACACTGCACCCTTGAC	Reverse
2.7	CTGGCCAACATCGTGAAACC	Forward
	AGCAATTCTCCTGACTCCGC	Reverse
20.7	TTTTTCCTGGTGAGGCAGGG	Forward
	CCTTGGAATTTCCGGGAGCT	Reverse

CHK1( kb relative to 1 exon)

**Table:** 

-1.1	CCTGTGGCTCGCTTCTGTAA	Forward
	AGACAGGGTGTGTTGCTCTG	Reverse
-0.6	CATACGCCTCAGCTTCCCAA	Forward
	AATGTTACTCAAGGCCGGGG	Reverse
0.1	GCAAAAAGACCGGGCTGAAG	Forward
	AGTTTCCCGGAGAAAGCGAG	Reverse
0.7	TCCACGTCACCCTTTTGGAG	Forward
	TCCAAATGCAGCGCTTTTCC	Reverse
1.6	TCCTGCCTTTTACAGCCGAG	Forward
	TGCACCAAGTCCCAGTCTTC	Reverse
3.4	CGAAGGACCTCACAGGCATT	Forward
	ATTGCAGGCATGTACCACCA	Reverse

### Immunostaining

Cells were fixed, permeabilized, and blocked as in flow cytometry analysis. Primary and secondary antibodies were incubated for 1hr each at RT. Anti-phospho-Histone H2AX (Ser139) (Millipore, #05-636), HP1α (Cell Signaling, #2616), tri-methyl Histone H3 (Lys4) (Cell Signaling, #9727), and H3K9me3 (Active Motif, #39285) were diluted in 10% goat serum/PBS and used at 1:500, 1:400, 1:2000, and 1:80 dilutions, respectively. AlexaFluor 488 conjugated-goat anti-rabbit (A11034) and AlexaFluor 555 conjugated-goat anti-mouse (A21424) antibodies (Molecular Probes) were used at 1:500 dilutions. Samples were mounted with Prolong® Gold Antifade Reagent with DAPI (Molecular Probes, P36935). Image was captured using a Zeiss LSM 510 confocal system using 63×1.4NA Plan-Apochromat oil immersion objective (Carl Zeiss, Inc., Thornwood, NY). Experiments were repeated and multiple fields were analyzed, containing 10-40 cells each.

### Accession numbers

The expression array data derived from BRD4 knockdown and JQ1-treated Ovcar8 cells are deposited in the Gene Expression Omnibus database under accession number GSE78704.

### Statistical comparisons

Statistical comparisons for Figure 1, between conditions of siNeg compared to siBRD4, used one-way ANOVA with Tukey post-hoc analysis to isolate the effect of siRNA on cell viability. Similar tests were carried out in conditions with or without JQ1. We used one-way ANOVA with Dunnett post-hoc adjustment for data in Figures 3C, 3D and 5E in order to focus on the differences of the experimental values to the negative control condition. For Figures 5C, 5F, 5H, 5I and 5K, ANOVA with Tukey post-hoc was used in order to correct for multiple comparisons between groups.

## SUPPLEMENTARY MATERIALS FIGURES AND TABLES






