# Supplementary material for: BRD4 facilitates DNA damage response and represses CBX5/Heterochromatin protein 1 (HP1)
Source: Oncotarget. 2017 May 3;8(31):51402–15. doi: 10.18632/oncotarget.17572 (PMC5584257; doi:10.18632/oncotarget.17572)
Supplement: Supplementary file 1 [file oncotarget-08-51402-s001.pdf]

BRD4 facilitates DNA damage response and represses CBX5/ Heterochromatin protein 1 (HP1)

SUPPLEMENTARY MATERIALS

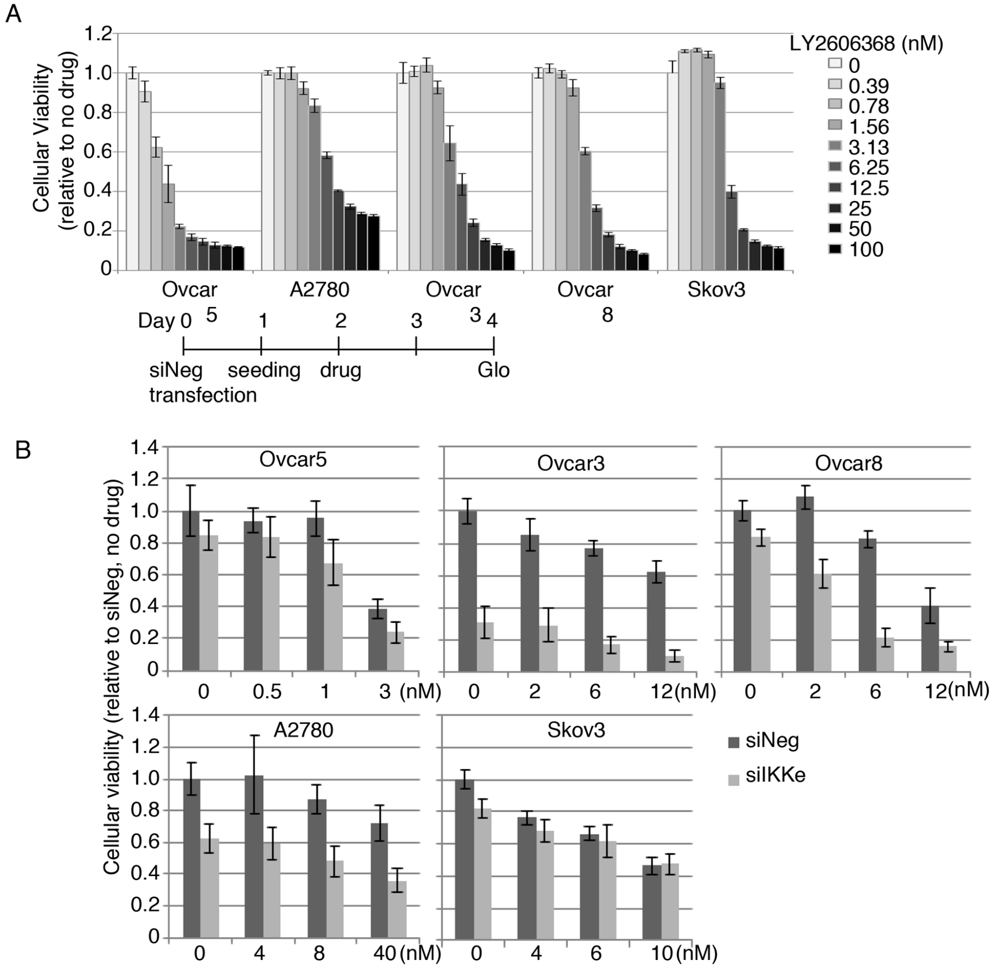

C

	LY2606368 (nM)	
	IC25	IC50
Ovcar5	1.5	3
Ovcar3	6	12
Ovcar8	5	10
A2780	10	40
Skov3	4	10

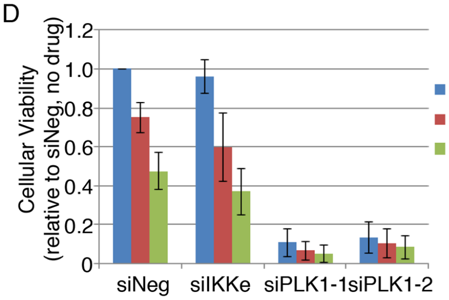

Supplementary Figure 1: Optimization of CHK1 inhibitor sensitization screen. (A) The time line of transfection and drug treatment was shown. Viability was calculated relative to no drug treatment; error bars represent standard deviation of 3 replicates. **(B)** The concentration for LY2606368 to reach IC25 and IC50 in each cell line was determined using control siRNAs **(C)** The concentrations for LY2606368 used in each screen are shown. **(D)** The average cellular viabilities from all cell lines using two positive siRNA controls were shown.

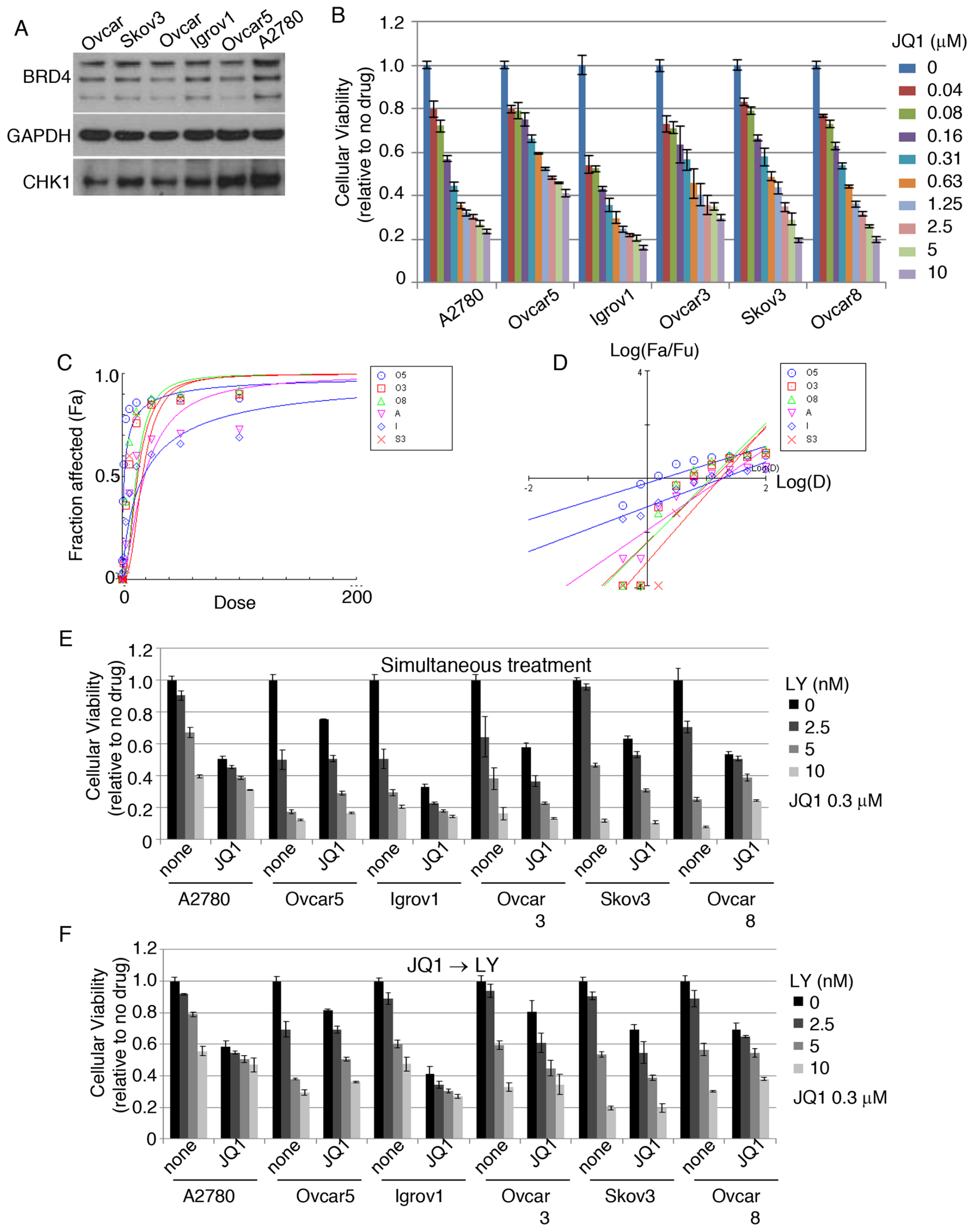

Supplementary Figure 2: BRD4 expression level and JQ1 toxicity in OC. **(A)** Total lysates were analyzed for BRD4 and CHK1 expression levels. GAPDH was used as a loading control. **(B)** Cells were seeded 24hr prior to adding JQ1. Viability was measured 72 hours after drug addition. **(C)** Fraction affected curve generated by Compusyn software based on the individual data points from (B). **(D)** Median dose effect plot for JQ1 in ovarian cancer cell lines (see Methods for calculation). **(E)** Cells were seeded 24hr prior to adding LY2606368 and JQ1. Viability was measured 72hr after drug addition. **(F)** Cells were seeded 24hr prior to adding JQ1 and then LY2606368 was added next day. Viability was measured 48hr after LY2606368 treatment.

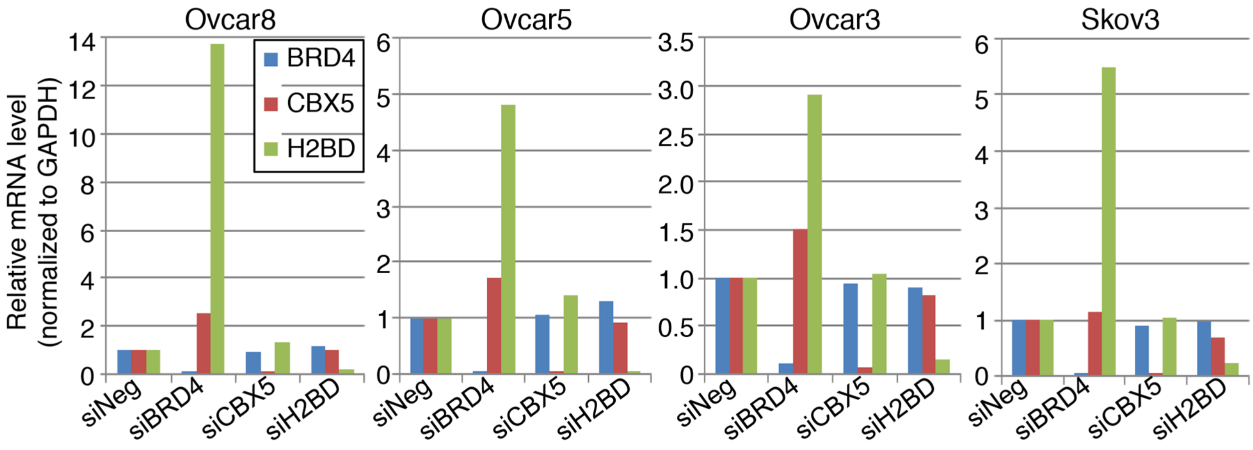

Supplementary Figure 3: Validation of siRNA knockdown. Total RNA was isolated at 48 hour post transfection and genes were validated by qPCR. Each gene was normalized by GAPDH and compared to negative control.

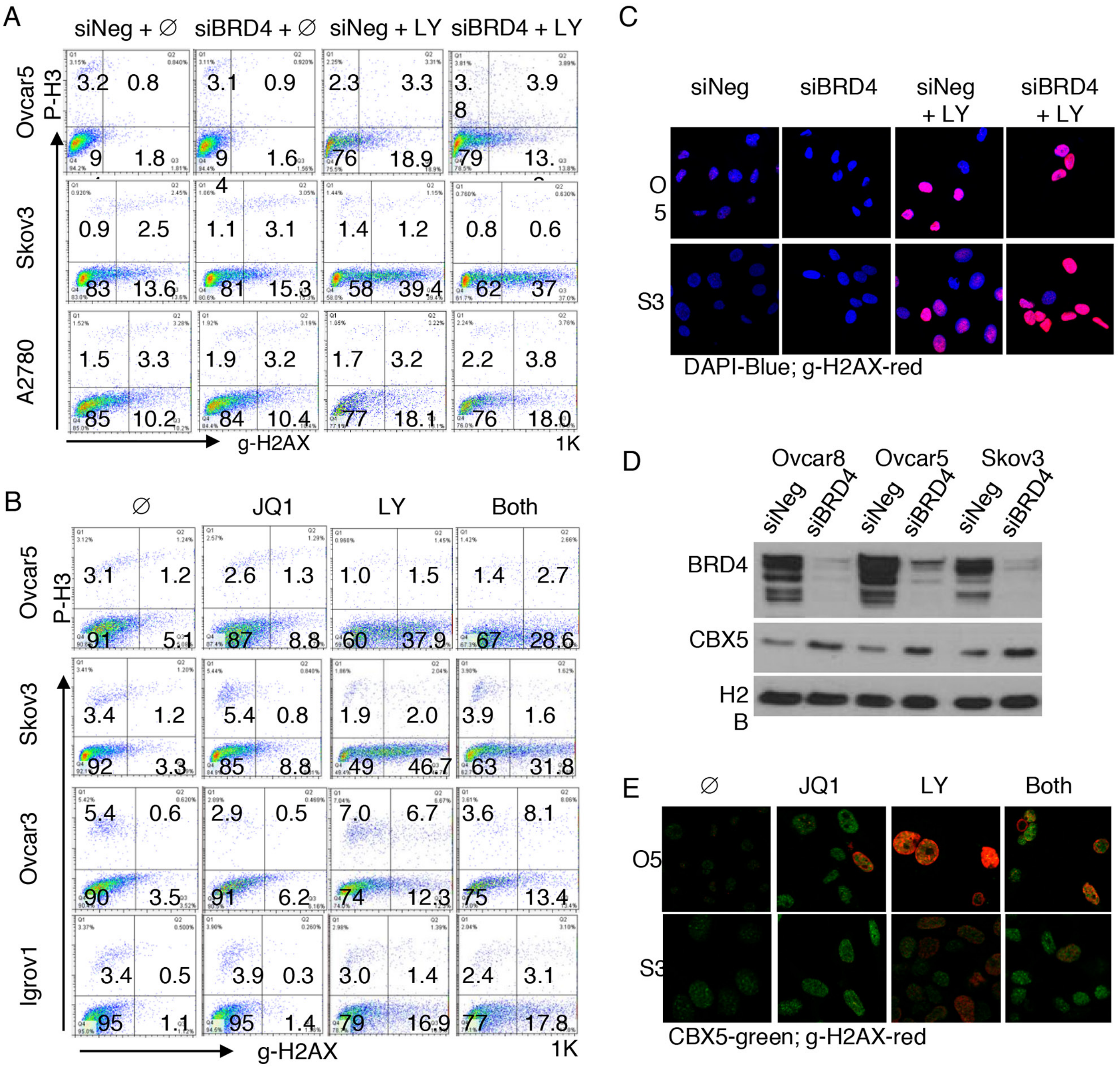

Supplementary Figure 4: BRD4 suppression induces heterochromatin, inhibiting DNA damage response. (A-C) Cells were transfected with either siNeg or siBRD4; 24hr later the cells were treated with LY for 20hr. For chemical inhibition, cells were treated with JQ1 and/or LY for 20hr and analyzed by flow cytometry (A-B), or for 30hr in immunofluorescent staining (C). **(D)** Nuclear lysates of siRNA transfected cells were prepared at 48 hour post transfection. H2B was used as a loading control. **(E)** Immunofluorescent staining of CBX5 and γ-H2AX were performed at 30hr post treatment. (*Continued*)

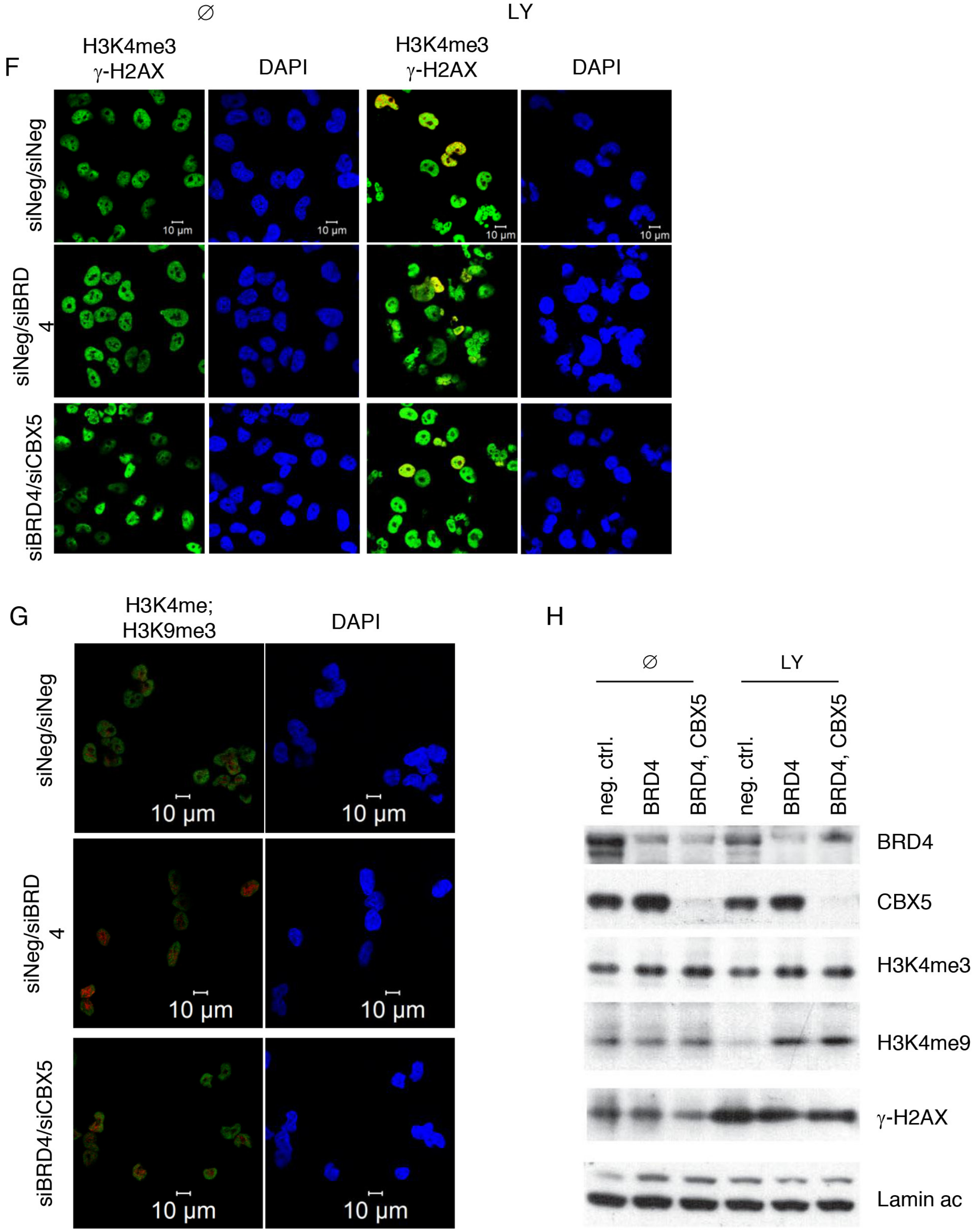

Supplementary Figure 4: (*Continued*) BRD4 suppression induces heterochromatin, inhibiting DNA damage response. **(F)** Using the same samples as shown in Figure 4F, the images were displayed as merged images of H3K4me3 and γ-H2AX, and DAPI separately. **(G)** Cells were co-stained with H3K4me3 and H3K9me3 antibodies at 72 hours post-transfection. **(H)** Ovcar8 cells were transfected with indicated siRNA constructs; after 24 hours, LY (10nM) was added and cells incubated additional 24 hours. Nuclear lysates were extracted and separated by electrophoresis. Western blot was performed with the indicated antibodies. Lamin-ac was used as a loading control.

Supplementary Table 1: siRNA values.

See Supplementary File 1

Supplementary Table 2: Differentially expressed genes.

See Supplementary File 2
